# Seropositivity and Risk Factors Associated with *Toxoplasma gondii* Infection in Wild Birds from Spain

**DOI:** 10.1371/journal.pone.0029549

**Published:** 2011-12-22

**Authors:** Oscar Cabezón, Ignacio García-Bocanegra, Rafael Molina-López, Ignasi Marco, Juan M. Blanco, Ursula Höfle, Antoni Margalida, Esther Bach-Raich, Laila Darwich, Israel Echeverría, Elena Obón, Mauro Hernández, Santiago Lavín, Jitender P. Dubey, Sonia Almería

**Affiliations:** 1 Servei d'Ecopatologia de Fauna Salvatge, Departament de Medicina i Cirurgia Animals, Facultat de Veterinaria, Universitat Autònoma de Barcelona, Bellaterra, Spain; 2 Departamento de Sanidad Animal, Facultad de Veterinaria, Universidad de Córdoba, Córdoba, Spain; 3 Centre de Fauna Salvatge de Torreferrussa, Direcció General del Medi Natural i de la Biodiversitat-Forestal Catalana, Generalitat de Catalunya, Santa Perpètua de la Mogoda, Barcelona, Spain; 4 Centro de Estudios de Rapaces Ibéricas, Sevilleja de la Jara, Castilla-la-Mancha, Spain; 5 Instituto de Investigación en Recursos Cinegéticos (IREC) (Centro Superior de Investigaciones Científicas-Universidad de Castilla-La Mancha-Junta de Castilla-La Mancha), Ciudad Real, Spain; 6 Bearded Vulture Study and Protection Group, El Pont de Suert, Lleida, Spain; 7 Division of Conservation Biology, Institute of Ecology and Evolution, University of Bern, Bern, Switzerland; 8 Centre de Recerca en Sanitat Animal (CReSA), Universitat Autònoma de Barcelona, Bellaterra, Barcelona, Spain; 9 Departament de Sanitat i d'Anatomia Animals, Facultat de Veterinària, Universitat Autònoma de Barcelona, Bellaterra, Spain; 10 Clínica Veterinaria Oftalmológica (OCUVET), Torrelodones, Madrid, Spain; 11 Animal Parasitic Diseases Laboratory, Animal and Natural Resources Institute, Agriculture Research Service, United States Department of Agriculture, Beltsville, Maryland, United States of America; Charité, Campus Benjamin Franklin, Germany

## Abstract

*Toxoplasma gondii* is a zoonotic intracellular protozoan parasite of worldwide distribution that infects many species of warm-blooded animals, including birds. To date, there is scant information about the seropositivity of *T. gondii* and the risk factors associated with *T. gondii* infection in wild bird populations. In the present study, *T. gondii* infection was evaluated on sera obtained from 1079 wild birds belonging to 56 species (including *Falconiformes* (n = 610), *Strigiformes* (n = 260), *Ciconiiformes* (n = 156), *Gruiformes* (n = 21), and other orders (n = 32), from different areas of Spain. Antibodies to *T. gondii* (modified agglutination test, MAT titer ≥1∶25) were found in 282 (26.1%, IC_95%:_23.5–28.7) of the 1079 birds. This study constitute the first extensive survey in wild birds species in Spain and reports for the first time *T. gondii* antibodies in the griffon vulture (*Gyps fulvus*), short-toed snake-eagle (*Circaetus gallicus*), Bonelli's eagle (*Aquila fasciata*), golden eagle (*Aquila chrysaetos*), bearded vulture (*Gypaetus barbatus*), osprey (*Pandion haliaetus*), Montagu's harrier (*Circus pygargus*), Western marsh-harrier (*Circus aeruginosus*), peregrine falcon (*Falco peregrinus*), long-eared owl (*Asio otus*), common scops owl (*Otus scops*), Eurasian spoonbill (*Platalea leucorodia*), white stork (*Ciconia ciconia*), grey heron (*Ardea cinerea*), common moorhen (*Gallinula chloropus*); in the International Union for Conservation of Nature (IUCN) “vulnerable” Spanish imperial eagle (*Aquila adalberti*), lesser kestrel (*Falco naumanni*) and great bustard (*Otis tarda*); and in the IUCN “near threatened” red kite (*Milvus milvus*). The highest seropositivity by species was observed in the Eurasian eagle owl (*Bubo bubo*) (68.1%, 98 of 144). The main risk factors associated with *T. gondii* seropositivity in wild birds were age and diet, with the highest exposure in older animals and in carnivorous wild birds. The results showed that *T. gondii* infection is widespread and can be at a high level in many wild birds in Spain, most likely related to their feeding behaviour.

## Introduction

*Toxoplasma gondii* is a zoonotic intracellular protozoan parasite of worldwide distribution [1], [2]. Wild and domestic felids are the definitive hosts, excreting oocysts in faeces. Humans and virtually all warm-blooded species, including birds, can be intermediate hosts and can become infected by ingestion of food and water contaminated with sporulated *T. gondii* oocysts, by consumption of tissue cysts in infected animal tissues, or congenitally [1], [2].

*Toxoplasma gondii* infection is prevalent in many avian species including poultry, game and other species in the wild [3], [4], [5], [6], [7], [8], [9]. In addition, clinical cases have been reported and *T. gondii* considered to be the cause of mortality in birds of different species [3], [4], [5], [10], [11]. However, there is still only scant information about seroprevalence of *T. gondii* and the risk factors associated to *T. gondii* infection in wild bird populations. Investigation of *T. gondii* seropositivity in birds could be a useful way to assess environmental contamination with oocysts since some avian populations feed directly on the ground and are continuously exposed to oocyst ingestion [11]. Furthermore, the investigation of *T. gondii* infection in avian scavengers might allow the assessment of the possibility of contact with intermediate hosts and the associated risk for public health [12].

In Europe, recent studies of *T. gondii* seropositivity have shown a high prevalence of *T. gondii* antibodies in wild birds. Aubert et al [4] reported prevalence levels of 79% of 14 common buzzards (*Buteo buteo*), 50% of 12 tawny owls (*Strix aluco*) and 11% of 18 barn owls (*Tyto alba*) in raptors from France. In Portugal, Lopes et al [6] reported a prevalence of antibodies in 50% of 52 wild birds that included multiple species such as black kite (*Milvus migrans*), booted eagle (*Hieraaetus pennatus*), common buzzard, Eurasian sparrowhawk (*Accipiter nisus*), Northern goshawk (*Accipiter gentilis*), Eurasian eagle owl (*Bubo bubo*) and tawny owl. Recently, in Spain, high seroprevalence of *T. gondii* in common ravens (*Corvus corax*) (80.5% of 113 sera samples) has been reported [7] and the presence of *T. gondii* DNA in brain tissues detected in magpies (*Pica pica*), Eurasian jays (*Garrulus glandarius*), black kite and griffon vulture (*Gyps fulvus*) [9].

The present study analyzed *T. gondii* seropositivity and provides information on possible associated risk factors, i.e. species, order, dietary habits, migration, sex, age and geographic origin in numerous species of wild birds from Spain, including some endangered species listed in the International Union for Conservation of Nature (IUCN) Red List of Threatened Species [13].

## Materials and Methods

### Ethics statement

Samples were collected in compliance with the Ethical Principles in Animal Research in the wildlife rehabilitation centres. The study of this material (blood serum) did not require the approval of an ethics committee because the collection of sera is considered among routine procedures and the sera was collected and stored before the design of this study. The rehabilitation centres directly depend on the governmental Autonomous Wildlife Services. Thus, protocols, amendments and other resources were done according to the guidelines approved by each Autonomous government following the R.D.1201/2005 (10^th^ October 2005, BOE 21^st^ October 2005) (http://www.umh.es/_web_rw/ceie/docs/animales/1201_05%20proteccion%20animales%20experimentacion.pdf) of the Ministry of Presidency of Spain.

### Samples

Serum samples from 1079 wild birds of 56 species (Table 1), belonging to the Orders *Falconiformes* (n = 610), *Strigiformes* (n = 260), *Ciconiiformes* (n = 156), *Gruiformes* (n = 21), and other orders (n = 32), were analyzed. The analyzed species included some listed on the IUCN Red List of Threatened Species [13] as near threatened [(*Aegypius monachus*, (n = 23), *Milvus milvus*, (n = 3), *Crex crex*, (n = 1)], as vulnerable [*Aquila adalberti* (n = 146); *Falco naumanni*, (n = 5), *Otis tarda*, (n = 7)] and; as endangered [*Neophron percnopterus*, (n = 49)].

**Table 1:** Seroprevalence of antibodies against *Toxoplasma gondii* (*T. gondii*) in wild birds from Spain using the modified agglutination test (MAT≥1∶25).

Species	IUCN-Red list	No. sera	% Positive
*Falconiformes^a^* *		602**	23.8
Griffon Vulture (*Gyps fulvus*)	Least Concern	175	17.71
Spanish Imperial Eagle (*Aquila adalberti*)	Vulnerable	146	17.07
Common Buzzard (*Buteo buteo*)	Least Concern	96	51.04
Egyptian Vulture (*Neophron percnopterus*)	Endangered	49	0.00
Cinereous Vulture (*Aegypius monachus*)	Near Threatened	23	26.09
Black Kite (*Milvus migrans*)	Least Concern	17	29.41
Bearded Vulture (*Gypaetus barbatus*)	Least Concern	15	42.86
Common Kestrel (*Falco tinnunculus*)	Least Concern	13	30.77
Short-toed Snake-eagle (*Circaetus gallicus*)	Least Concern	10	50.00
Bonelli's Eagle (*Aquila fasciata*)	Least Concern	9	11.11
Golden Eagle (*Aquila chrysaetos*)	Least Concern	8	62.50
Osprey (*Pandion haliaetus*)	Least Concern	7	28.57
Montagu's Harrier (*Circus pygargus*)	Least Concern	7	14.29
Eurasian Sparrowhawk (*Accipiter nisus*)	Least Concern	7	0.00
Western Marsh-harrier (*Circus aeruginosus*)	Least Concern	6	50.00
Northern Goshawk (*Accipiter gentilis*)	Least Concern	5	40.00
Lesser Kestrel (*Falco naumanni*)	Vulnerable	5	40.00
Peregrine Falcon (*Falco peregrinus*)	Least Concern	4	25.00
European Honey-buzzard (*Pernis apivorus*)	Least Concern	3	0.00
Red Kite (*Milvus milvus*)	Near Threatened	3	33.33
Northern Harrier (*Circus cyaneus*)	Least Concern	1	0.00
Booted Eagle (*Hieraaetus pennatus*)	Least Concern	1	0.00
***Strigiformes^b^***		**259** **	**44.0**
Eurasian Eagle-owl (*Bubo bubo*)	Least Concern	144	68.06
Barn Owl (*Tyto alba)*	Least Concern	45	13.33
Tawny Owl (*Strix aluco*)	Least Concern	38	13.16
Little Owl (*Athene noctua*)	Least Concern	19	15,79
Long-eared Owl (*Asio otus*)	Least Concern	9	11.11
Common Scops-owl (*Otus scops*)	Least Concern	4	25.00
Short-eared Owl (*Asio flammeus*)	Least Concern	1	0.00
***Ciconiiformes^c^***		**154** **	**11.0**
Eurasian Spoonbill (*Platalea leucorodia*)	Least Concern	81	6,17
White Stork (*Ciconia ciconia*)	Least Concern	64	14.06
Grey Heron (*Ardea cinerea*)	Least Concern	5	60.00
Purple Heron (*Ardea purpurea*)	Least Concern	4	0.00
Black Stork (*Ciconia nigra*)	Least Concern	1	0.00
Cattle Egret (*Bubulcus ibis*)	Least Concern	1	0.00
***Gruiformes^ac^***		**18** **	**5.6**
Red-knobbed Coot (*Fulica cristata*)	Least Concern	11	0.00
Great Bustard (*Otis tarda*)	Vulnerable	7	14.29
Corncrake (*Crex crex*)	Near Threatened	1	0.00
Common Coot (*Fulica atra*)	Least Concern	1	100.00
Common Moorhen (*Gallinula chloropus*)	Least Concern	1	100.00
***OTHERS^ac^***			
***Charadriiformes***		**16** **	**12.5**
Eurasian Thick-knee (*Burhinus oedicnemus*)	Least Concern	6	0.00
***Passeriformes***			
Black-billed Magpie (*Pica pica*)	Least Concern	4	0.00
Eurasian Linnet (*Carduelis cannabina*)	Least Concern	3	0.00
European Goldfinch (*Carduelis carduelis*)	Least Concern	1	100.00
Eurasian Jackdaw (*Corvus monedula*)	Least Concern	1	0.00
Northern House-martin (*Delichon urbicum*)	Least Concern	1	0.00
European Serin (*Serinus serinus*)	Least Concern	1	0.00
***Anseriformes***			
Mallard (*Anas platyrhynchos*)	Least Concern	6	33.33
Northern Pintail (*Anas acuta*)	Least Concern	1	100.00
Red-crested Pochard (*Netta rufina*)	Least Concern	1	0.00
***Apodiformes, Columbiformes, Galliformes, Podicipediformes and Pelecaniformes***			
Common Pheasant (*Phasianus colchicus, Galliformes*)	Least Concern	2	0.00
Eurasian Collared-dove (*Streptopelia. decaocto, Columbiformes*)	Least Concern	1	0.00
Great Cormorant (*Phalacrocorax carbo, Pelecaniformes*)	Least Concern	1	0.00
Little Grebe (*Tachybaptus ruficollis, Podicipediformes*)	Least Concern	1	0.00
Common Swift (*Apus apus, Apodiformes*)	Least Concern	1	0.00
Eurasian Hoopoe (*Upupa epops, Apodiformes*)	Least Concern	1	0.00

*Different letters among orders indicate statistically significant differences.

**only those species with more than 3 analyzed samples (birds) were included in the statistical analysis.

Samples were collected from wild birds captured or brought to wildlife rehabilitation centres and other breeding centres between 1996 and 2010. Blood collection was from the jugular vein, the heart, or the brachial vein. Blood was placed in a serum collection tube until clotted and then centrifuged. Sera were stored at −20°C until analysis. The samples were obtained from the main geographic areas from Spain including Northern Spain (n = 332) (Catalonia (228 samples) and Castile and León (4 samples from Valladolid); Central Spain (Castile La Mancha) (n = 565); and Southern Spain (Andalusia) (n = 282). The provinces sampled within Catalonia were Lleida and Barcelona; within Castile-La Mancha the provinces of Toledo and Ciudad Real, and within Andalusia the provinces of Granada, Málaga, Cádiz, Huelva, Seville, Córdoba and Jaén.

### Serological test

Sera were examined by the modified agglutination test (MAT) to detect antibodies against *T. gondii* as described previously [14]. Sera were tested at 1∶25, 1∶50, 1∶100 and 1∶500 dilutions. A commercial positive control (Toxotrol-A, Biomerieux, France) diluted from 1∶25 to 1∶3200 (with a minimum titre of 1∶200) was included in each test. Negative controls were also included in all tests. Titres of 1∶25 or higher were considered positive and those with doubtful results were re-examined. This technique has been previously evaluated in several bird species [3], [15].

### Definition of variables and Statistical analysis

Bird species were classified in regard to feeding behaviour as carnivorous (birds of prey), scavenger (when they feed on dead or decaying matter), granivorous-herbivorous-insectivorous (G/H/I), omnivorous and piscivorous. Whenever possible, sex and age data were collected. Age was classified in two categories (<1 year and ≥1 year). In addition, wild birds were classified as migratory or sedentary (non-migratory), according to their movements [16].

The variables analyzed included wild bird species, order, feeding behavior, geographic area of sample collection, migratory behavior, sex and age. For all statistical analysis only those species with more than 3 analyzed samples (birds) were included.

Association between explanatory variables and *T. gondii* seropositivity was tested in three steps. Firstly, a bivariate chi-square test was performed to obtain an indication of the relevance of the explanatory variables to the risk of an animal being seropositive. When observations per category were less than six, Fisher's exact test was used. Secondly, factors showing a *p*-value<0.25 were further scrutinized for associations using Spearman's rank correlation coefficient (*r*) to avoid colinearity problems. If *r* was larger than 0.4 the variable more clearly linked to *T. gondii* infection, based on epidemiological data and previous studies in the literature, was retained. The third step involved a multiple logistic-regression model using a non-automatic backward selection of variables. Biologically plausible confounding factors were tested using Mantel-Haenszel analysis and confounding was considered to be potentially significant if ORs shifted appreciably. Changes in the OR greater than 30% were considered indicative of confounding. The fit of the models was assessed using the Hosmer and Lemeshow goodness-of-fit test [17]. Potential two-way interactions between the variables were tested for significance in the model. The model was re-run until all the remaining variables presented statistically significant values (likelihood-ratio Wald's test, *p*<0.05), and a potential causal relationship with the response variable existed. Statistical analyses were performed using SPSS 15.0 (Statistical Package for Social Sciences (SPSS) Inc., Chicago, IL, USA).

## Results

The overall seropositivity (MAT≥1∶25) against *T. gondii* was 26.1% (CI_95%_: 23.5–28.7) (282 positive of 1079 wild birds tested), with titres of 1∶25 in 145 samples (51.4%), 1∶50 in 112 samples (39.7%), 1∶100 in 13 samples (4.6%) and 1∶500 in 12 samples (4.3%). The results by analyzed species are shown in Table 1.

In the bivariate analysis, considering those species with more than 3 samples analyzed, statistically significant differences were observed between seropositivity levels and the variables: order, wild bird species, geographic area of sample collection, migratory behaviour, age, year of sampling and feeding behaviour.

Based on their feeding behaviour, carnivorous wild birds showed statistically significant higher seropositivity of *T. gondii* (36.2% of 588) compared to scavenger (15.6% of 262 samples), omnivorous (13.2% of 68), G/H/I (11.8% of 34), and piscivorous (10.3% of 97) (*P<*0.001) species.

The order *Strigiformes* showed statistically higher seropositivity (44.0% of 259) compared to all the other orders (*P<*0.01). In addition, the seropositivity in the order *Falconiformes* (23.8% of 602) was significantly higher than that in the order *Ciconiiformes* (11% of 154) (*P<*0.05). Seropositivity in *Gruiformes* order was 5.6% (1 of 18), and in other orders was 12.5% (2 of 16).

The highest seropositivity by species was observed in the Eurasian eagle owl 68.1% of144 samples. A higher than 50% seropositivity was observed in: golden eagle (*Aquila chrysaetos*) (62.5% of 8), grey heron (*Ardea cinerea*) (60% of 5) and, in common buzzard (*Buteo buteo*) (51% of 96). Interestingly, no antibodies were observed in the endangered Egyptian vulture (*N. percnopterus*) (0 of 49 samples). Statistically significant differences were observed between *B. bubo* and *B. buteo* compared to *A. adalberti*, *Ciconia ciconia, Fulica cristata, G. fulvus*, *N. percnopterus, Platalea leucorodia, S. aluco and T. alba* (*P<*0.05). In addition, statistically significant differences were observed betwen *B. bubo* compared to *A. nisus*, *A. monachus*, *Gypaetus barbatus*, and *Aquila fasciata* (*P<*0.05).

The positivity of infection was significantly higher (*P<*0.001) in samples collected from wild birds from Northern (mostly Catalonia) and Central Spain (27.2 and 30.2%, respectively) compared to Southern Spain (18.3%), (*P<*0.001).

Sedentary wild birds had significantly higher seropositivity (31.1% of 795 samples) compared to migratory birds (12.0% of 249) (*P<*0.001).

**Figure 1:**
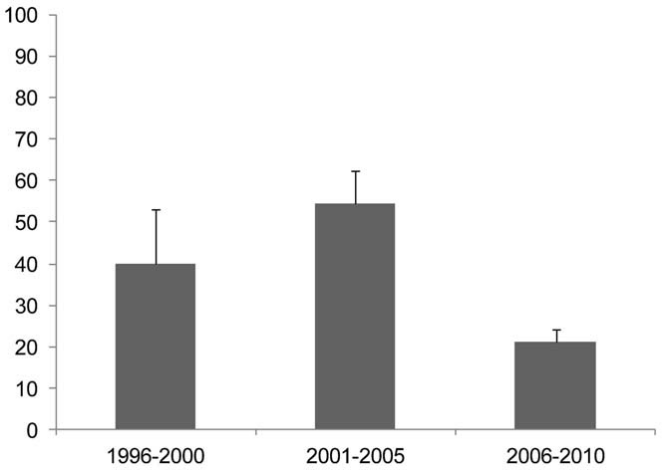
Seropositivity of *Toxoplasma gondii* (95% confidence limits) in wild birds from Spain among periods (years) of sampling.

Significant differences were observed between samples from birds older than 1 year showed statistically significant higher seropositivity (38.6% of 158) than birds younger than 1 year (9.5% of 148). Significant differences were also observed also among years of sampling (*P<*0.001). Samples collected up to the year 2000 showed high seropositivity but the number of analyzed samples was low (mean of the 1996–2000 period: 40.0% of 55 samples), (Figure 1). The period of highest seropositivity was observed between 2001 and 2005 (mean of the 2001–2005 period, 54.6% of 152 samples), then seropositivity decreased from 2006 onward maintaining a similar level up to 2010 (mean of the period 2006–2010, 21.2% of 641 samples) (Figure 1). Seropositivity in the latter period was significantly lower compared to the other two periods (P<0.01).

The final multivariate model showed that the main risk factors associated with *T. gondii* seropositivity in wild birds were age and feeding behaviour. Older wild birds had a 6.8 times higher risk of being seropositive compared to younger birds (>1 year compared to <1 year birds (OR = 6.8; 95% CI = 2.71–17.29) and carnivorous wild birds had a 5.4 times higher risk compared to piscivorous wild birds (taken as reference) (OR = 5.36; 95% CI = 2.70–10.6) (Table 2).

**Table 2:** Logistic regression analysis of potential risk factors associated with *T. gondii* seropositivity in wild birds in Spain.

Variable	Category	*β*	*P*-value	OR	95% CI
Food	Carnivorous	1.68	<0.001	5.36	(2.69–10.68)
	Granivorous/Herbivorous/Insectivorous	−0.97	0.221	0.38	(0.08–1.78)
	Omnivorous	−18.86	0.999	0.00	
	Scavenger	0.71	0.231	2.04	(0.63–6.53)
	Piscivorous	*	*	*	*
Age	>1 year old	1.92	<0.001	6.85	(2.71–17.29)
	<1 year old	*	*	*	*

*Reference category.

## Discussion

The present study included a wide range of wild bird species and a high number of analyzed samples supplements the existing data on seropositivity of *T. gondii* in wild birds worldwide, and adds numerous new species of wild birds to the possible intermediate host list for *T. gondii* infection. To our knowledge, this is the first report of *T. gondii* antibodies in griffon vulture, short-toed snake-eagle, Bonelli's eagle, golden eagle, bearded vulture, osprey, Montagu's harrier, Western marsh-harrier, peregrine falcon, long-eared owl, common scops owl, Eurasian spoonbill, white stork, grey heron, common moorhen, the IUCN “vulnerable” Spanish imperial eagle, lesser kestrel and great bustard; and the IUCN “near threatened” red kite and it is the first survey of these wild bird species in Spain. Detection and/or isolation of the parasite from these species would be necessary to corroborate that these species are intermediate hosts of *T. gondii*.

The results showed that *T. gondii* infection is widespread in many wild birds in Spain, with high variation among different species, orders, geographical regions and feeding behaviour. The main risk factors associated with *T. gondii* seropositivity in wild birds were age and feeding behaviour, with higher exposure observed in older animals and in species with a meat-based diet. The observed age effect is in accordance with previous studies of *T. gondii* seroprevalence in other animal species such as pigs, Iberian lynx (*Lynx pardinus*), Spanish ibex (*Capra pyrenaica*) and cats in Spain [18], [19], [20], [21] as well as in numerous studies of other species worldwide [2], and would be an indication of cumulative likelihood for exposure to *T. gondii* with age [2], [22] and to lifelong persistence of antibodies. The higher seropositivity observed in older individuals could also be an indication of horizontal transmission as the main route of transmission of *T. gondii* in these species.

Carnivorous species showed a significantly higher seropositivity of *T. gondii.* In this respect, the order *Strigiformes* (in which virtually all the species are carnivorous) showed the highest seropositivity, followed by the order *Falconiformes*, in which the majority of species are carnivorous and/or scavenger species. The order *Gruiformes,* with mostly G/H/I species, showed the lowest seropositivity. This agrees with the results obtained in mammals, in which several studies have observed that carnivorous species have higher seroprevalence of *T. gondii* antibodies compared to omnivorous, herbivorous or insectivorous species [23], [24], [25], [26]. A possible explanation could be the cumulative ingestion of infected prey, as suggested by similar results from comparison of seroprevalence levels in carnivorous mammals such as cats [21], omnivorous such as wild boars [27] and herbivorous such as red deer and other wild ruminants [28] in Spain. The main mode of infection of wild birds, as of other intermediate hosts, with *T. gondii* is through ingestion of food and water contaminated with sporulated oocysts (mainly in ground feeding birds) and by consumption of cysts contained in tissues of infected animals (mainly in carnivorous, scavenger birds) [2]. The present results suggest that the main mode of transmission of *T. gondii* in the analyzed wild birds was consumption of tissue cysts.

In agreement with the above results, the highest seropositivity was observed in the Eurasian eagle owl (68.1% of 144), a carnivorous species recently included in the list of birds with *T. gondii* antibodies by Lopes et al [6]. High seropositivity was also observed in other carnivorous species such as the common buzzard, which has been previously reported to have high seroprevalence in France (79% of 14 samples) [4], and in the golden eagle. The main diet of these species of carnivorous wild birds includes rodents and other small mammals, which are well known reservoirs of *T. gondii* infection for many animal species, especially pigs [25], [29], [30]. On the other hand, high seropositivity was also observed in some omnivorous species, such as the grey heron (60% of 5) and in some species of duck [*Anas acuta* (100% of 1) and *A. plathyrhynchos* (33.3% of 6)], although the number of analyzed samples was relatively low. The grey heron mainly feeds in shallow water catching fish, frogs, and insects, but will also eat small mammals, reptiles and other birds [31] and, like other *Ciconiiformes*, has been moving into urban environments where they also make use of food discarded by humans. The fact that grey heron samples were mainly from Catalonia (4 of 5 samples, with all the positive samples from this location), which seems to be one of the most prevalent areas of *T. gondii* infection in Spain, with higher prevalence of *T. gondii* compared to other locations for species such as wild rabbits [32], roe deer [33] and domestic pigs [18], and where higher detection of the parasite has also been observed in tissues from wild birds [9], could also be an explanation for the increased seropositivity observed in this species. Moderate precipitation and humidity in those areas might favour oocyst survival and sporulation in the environment facilitating *T. gondii* spread and maintenance [1]. The ducks analyzed in the present study were from different geographical regions, indicating that seroprevalence was not due to location. High seroprevalence in ducks has been recently reported by Alvarado-Esquivel et al [8] (2 positive samples, in 1 *A. platyrhynchos* and 1 *A. diazi*, of 4 ducks analyzed).

Similarly, it was also unexpected to observe high seropositivity of *T. gondii* in bearded vultures (*G. barbatus*) (42.9% of 15) because this species feeds primarily upon livestock bones. A possible explanation is that during the chick-rearing period, small and medium-sized mammals and other birds are also included in the chickś, and even in the parentś diet [34]. Furthermore, most bearded vultures were also obtained in the northern area. Further studies including more samples of this species will need to be performed to confirm this observation.

To the contrary, the Egyptian vulture, a small vulture inhabiting open landscapes in arid and rugged regions in the Mediterranean, Africa and Asia, did not show antibodies to *T. gondii*. The diet of Egyptian vultures is based on carcasses of small and medium-sized wild and domestic animals and, in a lesser degree, livestock, usually ingesting small pieces of skin or muscle [35] which could be the reason for the observed lack of contact with *T. gondii* in the analyzed samples.

Interestingly, some piscivorous birds showed contact with the parasite, including some species such as the Eurasian spoonbill (*P. leucorodia*) analyzed in high numbers, (6.2% of 81 samples). This result could be an indication of water contamination by oocysts. Until recently, waterborne transmission of *T. gondii* was considered uncommon. However, a large human outbreak linked to contamination of a municipal water reservoir in Canada by wild felids and the widespread infection of marine mammals in the USA provided reasons to question this view [36]. *Toxoplasma gondii* antibodies have been observed in Mediterranean wild dolphins [37] and a case of systemic toxoplasmosis in a pregnant dolphin from the Spanish Mediterranean coast has been reported [38].

In summary, the present study extends the range of species of wild birds that have antibodies to *T. gondii* and indicates widespread exposure to *T. gondii* among many wild bird species in Spain, with highly variable postitivity levels among species and orders. The main risk factors associated with *T. gondii* seropositivity in wild birds were age and feeding behaviour, with higher exposure in older animals and in carnivorous wild birds. The results indicate that *T. gondii* infection can be high in some species, mainly related to their feeding behaviour, and that these species may have an important role in the epidemiology of *T. gondii* infection. The observed contact with the parasite in some vulnerable or near threatened wild bird species suggests that the analysis of this parasite should be taken into account in relevant wild bird conservation programs.
